# Machine Learning Assessment of Early Life Factors Predicting Suicide Attempt in Adolescence or Young Adulthood

**DOI:** 10.1001/jamanetworkopen.2021.1450

**Published:** 2021-03-12

**Authors:** Marie C. Navarro, Isabelle Ouellet-Morin, Marie-Claude Geoffroy, Michel Boivin, Richard E. Tremblay, Sylvana M. Côté, Massimiliano Orri

**Affiliations:** 1 Bordeaux Population Health Research Center, Institut national de la santé et de la recherche médicale U1219, University of Bordeaux, Bordeaux, France; 2 School of Criminology, Research Center of the Montreal Mental Health University Institute, University of Montreal, Montreal, Canada; 3 McGill Group for Suicide Studies, Department of Psychiatry, Douglas Mental Health University Institute, McGill University, Montreal, Canada; 4 School of Psychology, University of Laval, Quebec City, Canada; 5 School of Public Health, Physiotherapy and Sports Science, University College Dublin, Dublin, Ireland; 6 Department of Pediatrics and Psychology, University of Montreal, Montreal, Canada; 7 Department of Social and Preventive Medicine, University of Montreal, Montreal, Canada

## Abstract

**Importance:**

Although longitudinal studies have reported associations between early life factors (ie, in-utero/perinatal/infancy) and long-term suicidal behavior, they have concentrated on 1 or few selected factors, and established associations, but did not investigate if early-life factors predict suicidal behavior.

**Objective:**

To identify and evaluate the ability of early-life factors to predict suicide attempt in adolescents and young adults from the general population.

**Design, Setting, and Participants:**

This prognostic study used data from the Québec Longitudinal Study of Child Development, a population-based longitudinal study from Québec province, Canada. Participants were followed-up from birth to age 20 years. Random forest classification algorithms were developed to predict suicide attempt. To avoid overfitting, prediction performance indices were assessed across 50 randomly split subsamples, and then the mean was calculated. Data were analyzed from November 2019 to June 2020.

**Exposures:**

Factors considered in the analysis included 150 variables, spanning virtually all early life domains, including pregnancy and birth information; child, parents, and neighborhood characteristics; parenting and family functioning; parents’ mental health; and child temperament, as assessed by mothers, fathers, and hospital birth records.

**Main Outcomes and Measures:**

The main outcome was self-reported suicide attempt by age 20 years.

**Results:**

Among 1623 included youths aged 20 years, 845 (52.1%) were female and 778 (47.9%) were male. Models show moderate prediction performance. The areas under the curve for the prediction of suicide attempt were 0.72 (95% CI, 0.71-0.73) for females and 0.62 (95% CI, 0.60-0.62) for males. The models showed low sensitivity (females, 0.50; males, 0.32), moderate positive predictive values (females, 0.60; males, 0.62), and good specificity (females, 0.76; males, 0.82) and negative predicted values (females, 0.75; males, 0.71). The most important factors contributing to the prediction included socioeconomic and demographic characteristics of the family (eg, mother and father education and age, socioeconomic status, neighborhood characteristics), parents’ psychological state (specifically parents’ antisocial behaviors) and parenting practices. Birth-related variables also contributed to the prediction of suicidal behavior (eg, prematurity). Sex differences were also identified, with family-related socioeconomic and demographic characteristics being the top factors for females and parents’ antisocial behavior being the top factor for males.

**Conclusions and Relevance:**

These findings suggest that early life factors contributed modestly to the prediction of suicidal behavior in adolescence and young adulthood. Although these factors may inform the understanding of the etiological processes of suicide, their utility in the long-term prediction of suicide attempt was limited.

## Key Points

**Question:**

Can early life factors (ie, in-utero, perinatal, infancy) be used to predict suicide attempt in adolescence or young adulthood?

**Findings:**

In this prognostic study of 1623 children from a representative longitudinal cohort study, random forest algorithms, including 150 potential factors, found that early life factors modestly contributed to the prediction of suicide attempt in adolescence or young adulthood, with 24% to 44% better prediction than chance. The most informative factors include birth-related characteristics, family and parents’ characteristics, parents’ mental health, and parenting practices.

**Meaning:**

These findings suggest that although early-life factors may contribute to understanding the etiological processes of suicide, their utility in the long-term prediction of suicide attempt was limited.

## Teaser

This prognostic study examines the use of early factors in predicting suicide attempt in adolescence and young adulthood.

## Introduction

Suicide is an important public health concern and the second leading cause of death among individuals aged 15 to 29 years.^1,2^ A history of suicide attempt is a main factor of completed suicide. Therefore, early identification of youth at risk for suicide attempt is critical to prevent suicide and to reduce negative health, social, and economic consequences.^3,4^ A number of studies have reported that proximal risk factors, such as bullying victimization,^5^ school performance,^6^ and cannabis and alcohol use,^7,8^ are important factors for adolescent suicidal behavior. However, there is increasing evidence suggesting that early life characteristics and exposures may have long-lasting influences on the risk of suicidal behavior. In line with the developmental origins of health and disease hypothesis,^9,10^ several epidemiological studies^11,12^ reported associations of a range of early life factors with suicidal behavior in the lifespan.^9,13^ These include socioeconomic factors (eg, family socioeconomic disadvantage, low parental education, low maternal age, and single parenthood at childbirth),^14,15,16,17,18^ exposure to substance in pregnancy (eg, maternal smoking),^19^ poor fetal growth (eg, low birth weight and fetal adversities),^14,15,20^ exposure to postnatal maternal depression^21^ and poor parent-child interactions during infancy.^22,23^ For example, a study using the Christchurch Health and Development Study reported that children of teenage mothers were 2-fold more likely to attempt suicide in adolescence compared with children of older mothers.^18^ In another study using the Québec Longitudinal Study of Child Development and the Avon Longitudinal Study of Parents and Children, exposure to fetal adversities was associated with higher risk of attempting suicide by age 21 years.^15^ A meta-analysis showed that low birth weight was associated with higher risk of suicidal ideation, suicide attempt, and suicide mortality in the lifespan.^16^

However, owing to methodological limits of the statistical models used in prior studies (mainly regression models), only a small number of risk factors have been jointly evaluated. This contrasts with the current understanding of the etiological processes of mental disorders, argued to involve hundreds of endogenous and exogenous factors in dynamic and constant interaction across complex partially embedded networks.^24,25^ Additionally, although studies have identified several associations between a range of early risk factors and suicide attempt later in life, it is unclear to what extent these factors contribute to the prediction of suicide attempt. A statistical association quantifies the relation between 2 observed variables, whereas a predictive model identifies the most parsimonious number of variables enabling a good prediction of new observations.^26^ It is important to note that, even when associations are longitudinal (ie, there is temporal precedence of the exposure on the outcome) and strong (eg, have a large effect size), they do not inform on whether a given factor (or set of factors) is useful to predict a new observation.^27^

Although many studies have established associations of some early life risk factors with suicide attempt, we are unaware of studies investigating the predictive value of such risk factors and simultaneously considering a large number of potential factors. This is a limitation, because improving early prevention of suicide attempt relies on the capacity to accurately identify individuals more likely to attempt suicide later in life. This limitation is not only theoretical. In a 2017 meta-analysis^28^ analyzing all the risk factors identified to be associated with suicidal thoughts and behaviors in the last 50 years, none was found to reliably predict a future suicide attempt better than chance.

Machine learning is a promising approach to optimize the prediction of future outcomes.^29^ As most mental health disorders can be framed as classification problems (ie, distinguishing between individuals who are affected or symptomatic vs those who are not affected or asymptomatic, or between individuals who attempted suicide or did not), machine learning techniques have recently attracted the attention of mental health researchers with emerging fields of research, such as computational psychiatry.^30^ Machine learning techniques allow researchers to simultaneously consider hundreds of potential factors and determine, without prior assumptions, the most effective and parsimonious algorithm to predict a new observation.

Using data from a large 20-year population-based longitudinal study, the aim of this study was to test the extent to which we could predict suicidal attempt during adolescence and young adulthood using a large number of early life factors assessed with parental reports and hospital records. Findings could provide important information on the predictive ability of early life factors to identify individuals who will attempt suicide 2 decades later, complementing the available evidence from association studies.

## Methods

### Participants

Participants for this prognostic study came from the Québec Longitudinal Study of Child Development (QLSCD), a representative longitudinal population-based cohort. The protocol of the QLSCD was approved by the Institut de la Statistique du Québec, the institute that conducted the study, and the St-Justine Hospital Research Center ethics committees. Written informed consent was obtained from all participants. This study follows the Transparent Reporting of a Multivariable Prediction Model for Individual Prognosis or Diagnosis (TRIPOD) reporting guideline for prediction model development.

The QLSCD initially included 2120 singletons born in Québec, Canada in 1997 or 1998, selected from the Québec Birth Registry using a stratified random procedure. Children were regularly assessed from ages 5 months to 20 years.^31^ Owing to attrition, this study included 1623 participants (77.6% of the initial cohort) with at least 1 assessment of suicide attempt between ages 13 and 20 years (Table 1).

**Table 1.** Characteristics of the Participants Included in the Study Sample^a^

Characteristic	No. (%)			*P* value^b^
	Total (N = 1623)	Females (n = 845)	Males (n = 778)	
Low birth weight (<2500 g)	49 (3.0)	25 (3.0)	24 (3.1)	.53
Perceived difficult temperament, mean (SD)^c^				
Mother	2.7 (1.6)	2.7 (1.6)	2.7 (1.6)	.70
Missing	7 (0.4)	4 (0.5)	3 (0.4)	
Father	2.9 (1.5)	2.8 (1.5)	2.9 (1.5)	.23
Missing	244 (15.0)	120 (14.2)	124 (15.9)	
Positive interactions, mean (SD)^d^	9.0 (1.1)	9.0 (1.1)	9.0 (1.0)	.48
Missing	2 (0.1)	1 (0.1)	1 (0.1)	
Family socioeconomic status, mean (SD)^e^	0.1 (1.0)	0.1 (1.0)	0 (1.0)	.30
Missing	6 (0.4)	3 (0.4)	3 (0.4)	
Age at birth, mean (SD), y				
Mother	29.4 (5.2)	29.5 (5.1)	29.3 (5.2)	.24
Missing	1 (0.1)	0	1 (0.1)	
Father	32.3 (5.5)	32.1 (5.4)	32.4 (5.6)	.40
Missing	116 (7.1)	57 (6.7)	59 (7.6)	
Family functioning score, mean (SD)^f^	1.7 (1.4)	1.7 (1.4)	1.7 (1.5)	.91
Missing	11 (0.7)	8 (0.9)	3 (0.4)	
Nonintact family (single or blended)	327 (20.1)	176 (2.8)	151 (19.4)	.55
Missing	3 (0.2)	2 (0.2)	1 (0.1)	
Maternal smoking during pregnancy	401 (24.7)	218 (25.8)	183 (23.5)	.30
Missing	9 (0.6)	5 (0.6)	4 (0.5)	
Maternal mental health				
Depression, mean (SD)^g^	1.4 (1.3)	1.3 (1.3)	1.4 (1.4)	.37
Missing value	6 (0.4)	2 (0.2)	4 (0.5)	
Antisociality in adolescence score, mean (SD)^g^	0.8 (0.9)	0.8 (0.9)	0.9 (1.0)	.36
Missing	52 (3.20)	24 (2.8)	28 (3.6)	
Paternal mental health				
Depression, mean (SD)^g^	1.0 (0.9)	1.0 (0.9)	1.0 (0.9)	.22
Missing value	228 (14.0)	114 (13.5)	114 (14.7)	
Antisociality in adolescence score, mean (SD)^h^	0.7 (0.9)	0.6 (0.9)	0.7 (0.9)	.07
Missing	233 (14.4)	113 (13.4)	120 (15.4)	

^a^ Variables were measured when the child was aged 5 months. Data were compiled from the final master file of the Québec Longitudinal Study of Child Development (1998-2018), Gouvernement du Québec, Institut de la Statistique du Québec.

^b^ Comparison of sex-specific samples; *P* values are based on a χ^2^ test of independence for categorical variables and on a Wilcoxon test for continuous variables.

^c^ Assessed with 7 items (eg, “How easy or difficult is it for you to calm or soothe your baby when he/she is upset?”) from the Infant Characteristics Questionnaire,^32^ administrated to both parents. Scores range from 0 to 10, with higher scores indicating more difficult temperament.

^d^ Assessed with 5 items from the Parent Practices Scale,^33^ evaluating positive interactions between the mother and the child. Scores ranges from 0 to 10, with higher scores indicating high positive interactions.

^e^ Assessed with an aggregate of 5 items regarding parental educational level, parental occupation, and annual gross income (range, −3 to 3, centered at 0, with higher scores indicating higher socioeconomic status).

^f^ Assessed with 7 items (eg, do not get along well together) from McMaster Family assessment administered to the mother (*Ontario Child Health Study: Reliability and Validity of the General Functioning Subscale of the McMaster Family Assessment Device*^34^). Scores range from 0 to 10, with higher scores indicating lower family functioning.

^g^ Assessed using a short version of the Centre for Epidemiological Study Depression Scale.^35^ Scores range from 0 to 10, with higher scores indicating higher depressive symptoms.

^h^ Assessed with binary questions on 5 different conduct problems based on the DSM-IV criteria for conduct disorder and antisocial personality disorder.^36^ Scores range from 0 to 5, with higher scores indicating more antisocial behaviors.

### Assessment of Suicidal Attempt

At ages 13, 15, 17, and 20 years, adolescents who answered positively to the question “In the past 12 months, did you ever seriously think of attempting suicide?” were then asked “In the past 12 months, how many times did you attempt suicide?” (dichotomized as 0 vs ≥1).^37^ At age 20 years, lifetime suicide attempt was additionally assessed with the questions “In your lifetime, have you ever been to the emergency room (ER) because you tried to kill yourself?” and “In your lifetime, have you ever been hospitalized after trying to kill yourself?” Questionnaires were provided in French or English depending on respondent preference. Participants responding yes to any question were considered as having attempted suicide.^38,39^

### Assessment of Early Life Factors

We used a broad range of potential factors reported by parents when the child was aged 5 months, together with factors extracted from hospital birth records. These potential factors are presented in the eTable in the Supplement. We assessed 150 variables encompassing sociodemographic factors as well as child, family, parental, and neighborhood characteristics. Perinatal child characteristics included birth weight, prematurity, Apgar score, and neonatal hospitalization. Parenting and family functioning characteristics included positive maternal interactions, assessed with 5 items from the Parent Practices Scale,^33^ evaluating positive interactions between the mother and the child; family socioeconomic status, assessed with an aggregate of 5 items regarding parental educational level, parental occupation, and annual gross income; and family functioning, assessed with 7 items (eg, do not get along well together) from the McMaster Family assessment^34^ administered to the mother. Parental characteristics included parental age at childbirth, immigration status, and employment. Parental mental health and behavior included anxiety, depression (measured using a short version of the Centre for Epidemiological Study Depression Scale^35^), and antisocial behavior (assessed with binary questions on 5 different conduct problems based on the DSM-IV criteria for conduct disorder and antisocial personality disorder^36^). Child temperament was assessed with 7 items (eg, “How easy or difficult is it for you to calm or soothe your baby when he/she is upset?”) from the Infant Characteristics Questionnaire,^32^ administrated to both parents.

### Statistical Analysis

#### Random Forest Approach

We used a random forest algorithm, a nonparametric ensemble machine learning method that aims to find the most accurate combination of variables to predict a new observation.^40^ Random forests are well adapted to mental health prediction; first, they can be applied to classification or regression prediction, and often mental health issues can be framed as classification problem; second, categorical and continuous variables can be used jointly as predictors; third, they have been demonstrated to be a performant and reliable machine learning method.^41,42^ Random forests result from the aggregation of a set of decision trees, created with recursive bootstraps of the initial sample.^43^ For each decision tree, two-thirds of the sample was used to create the prediction algorithm, while the remaining one-third was used to test the performance of the algorithm, measured by the prediction error (called *out-of-bag error*) and to calculate the importance of the variables in the prediction (eAppendix in the Supplement). Decision trees proceed from a parent node to a child node, according to the optimal split value of the variable obtained according to the principle of maximum homogeneity for the outcome in each node. Derived trees are then aggregated to obtain the final prediction model. The synthetic minority over-sampling technique algorithm was used to avoid bias due to controls outnumbering cases.^44^ Previous studies reported that combining random forests and the synthetic minority over-sampling technique improve the prediction performances.^45,46^ The R statistical software version 4.0.2 (R Project for Statistical Computing) *missForest* algorithm was used to impute missing data in the factors (eAppendix in the Supplement).^47^ To perform the analysis, we randomly split our original data set into training (80% of the total cohort) and testing (20% of the total cohort) samples. The training samples were used to compute the predictive algorithms for the outcome.^48^ Preliminary analyses were conducted with sex as a factor in the models, but considering the important sex differences in suicide attempt,^4^ this variable overshadowed all other variables in terms of prediction and precluded us from investigating sex differences. Therefore, we conducted separate analyses for males and females. Analyses were performed in R statistical software with the *randomForest* and *caret* packages.

#### Evaluating Model Performance

Model performance (ie, the accuracy of the model in predicting new cases) was evaluated using out-of-bag error, defined as the prediction error obtained in the out-of-bag set using the identified factors (values ranged from 0%, indicating that all the individuals are correctly classified, to 100%, indicating that none of the individuals are correctly classified); area under the receiver operating characteristic curve (AUC),^49^ representing the predicted true-positive rate against the false-positive rate, which measures the accuracy of the prediction and ranges from 0.5, indicating prediction by chance, to 1, indicating perfect prediction; sensitivity, representing the proportion of actual cases that the model predicted to be cases, and specificity, representing the proportion of actual noncases that the model predicted as noncases; and positive predictive value (PPV), defined as the proportion of actual cases among those that the model predicted would be cases, and negative predictive value (NPV), defined as the proportion of actual noncases among those that the model predicted would be noncases. To obtain unbiased prediction performances, we first created the prediction algorithms in the training sample, containing 80% of the observations, and then tested its performance in the testing sample, containing the remaining 20% of observations. To prevent our prediction performances to be underestimated or overestimated owing to a particular random split of the sample, we randomly generated 50 training and testing samples, repeated the analyses 50 times, and reported the mean values of the predictive performances’ indices.

All statistical tests were 2-tailed, and the level of statistical significance was *P* < .05. Data were analyzed from November 2019 to June 2020.

## Results

**Figure 1.**
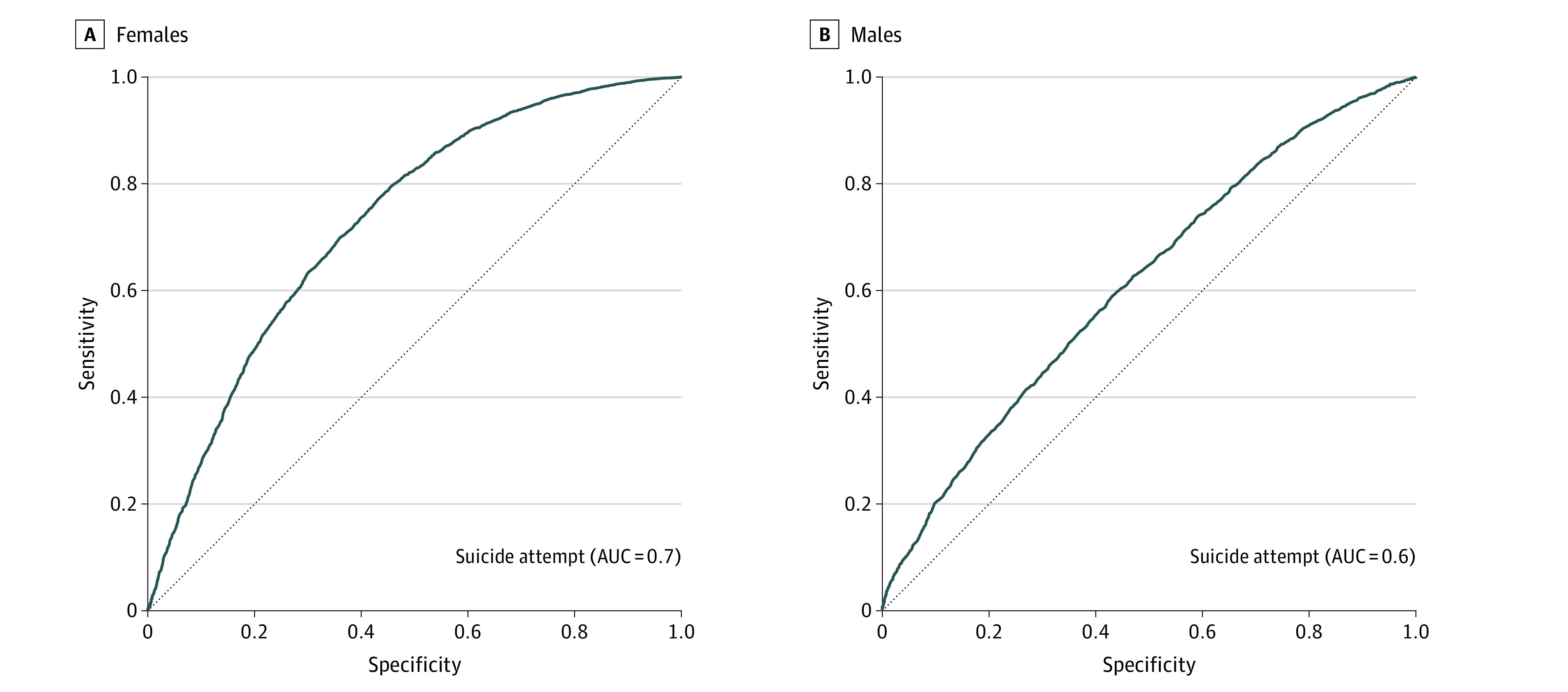
Area Under the Receiver Operating Curve of the Predictive Models of Lifetime Suicide Attempt

**Figure 2.**
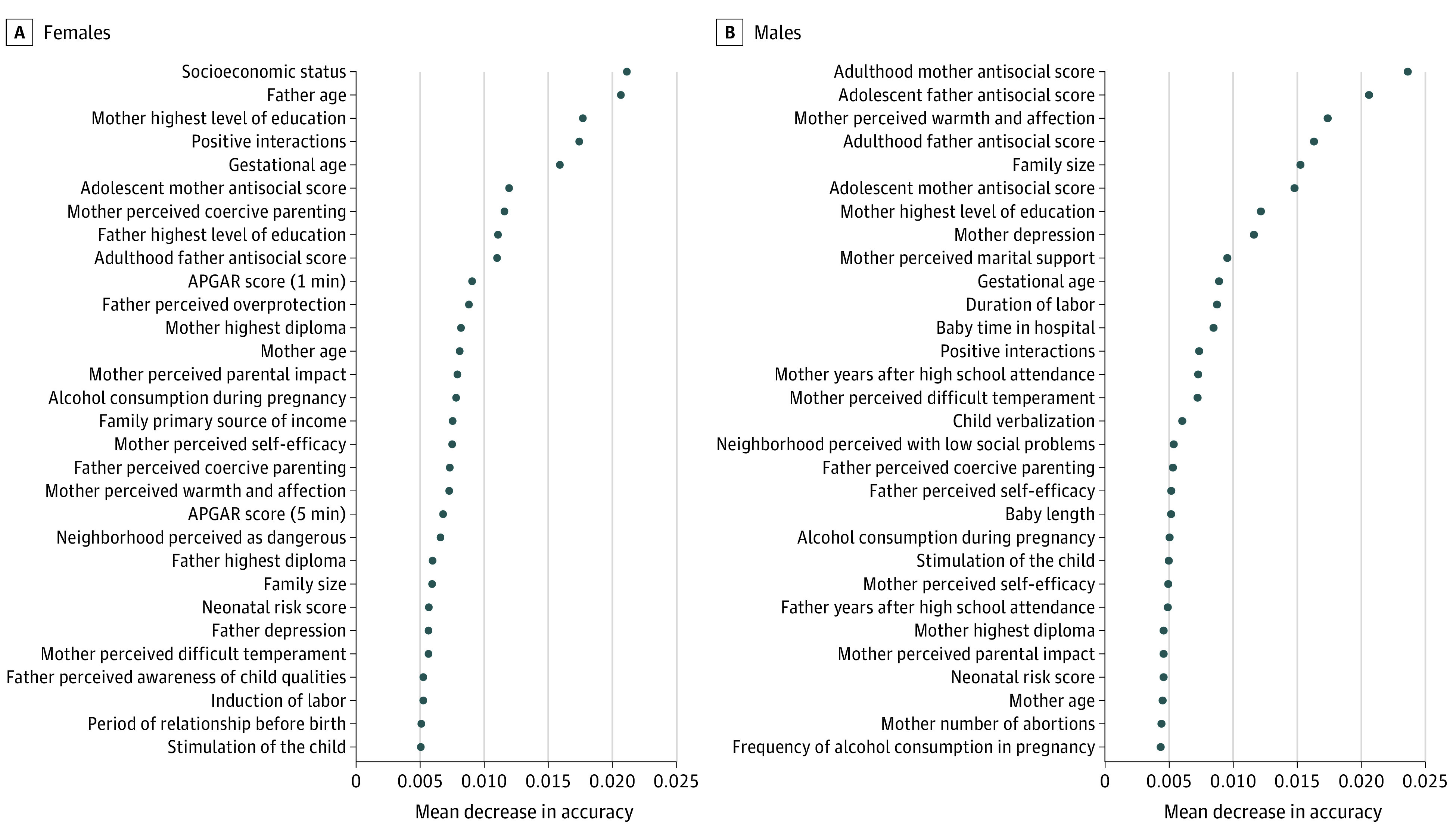
Relative Importance of the 30 Top Factors Identified by the Algorithm Predicting Suicide Attempt The relative importance of the early life factors considered in the random forest model is measured by the decrease in the prediction accuracy of the model (ie, mean decrease in accuracy, x-axis) when the variables’ values are randomly shifted in the model.

A total of 1623 participants were included in the sample, and 91 of 845 females (10.8%) and 43 of 778 (5.5%) of males reported a suicide attempt in adolescence. The random forest model predicting suicide attempt among females, obtained with the training sample, had an out-of-bag error of 12.7%, suggesting that only a small proportion of females were misclassified by the algorithm using the selected set of variables. When applied to the testing sample, this classification algorithm achieved a sensitivity of 0.50 and a specificity of 0.76. This suggests that the algorithm correctly identified as cases 50% of youths who attempted suicide and correctly predicted 76% of youths would not attempt suicide. The PPV was 0.60, suggesting that 60% of the individuals that the model identified as cases were actually cases. Similarly, the NPV was 0.75 indicating that 75% of youths that the model predicted would not attempt suicide were correctly identified. The AUC was 0.72 (95% CI, 0.71-0.73), reflecting a moderately good discrimination (ie, 44% better than chance) (Figure 1). Performance metrics are presented in Table 2. Variables’ importance, measured by mean decrease in accuracy of the prediction, showed that the top 10 early life factors of suicide attempt in females were socioeconomic status, father age, mother highest level of education, positive interactions, gestational age, adolescent mother antisocial score, mother perceived coercive parenting, father highest level of education, adulthood father antisocial score, and Apgar score at 1 minute (Figure 2).

**Table 2.** Discrimination Performances for the Best Prediction Models^a^

Measure	%	
	Females	Males
Out-of-bag error	12.7	9.3
Area under the curve (95% CI)	0.72 (0.71-0.73)	0.62 (0.60-0.62)
Sensibility	0.50	0.32
Specificity	0.76	0.82
Predictive value		
Positive	0.60	0.62
Negative	0.75	0.71

^a^ Data were compiled from the final master file of the Québec Longitudinal Study of Child Development (1998-2018), Gouvernement du Québec, Institut de la Statistique du Québec.

Similar to the model for females, the final prediction algorithm for males found only a small rate of misclassification (out-of-bag error of 9.3%) (Table 2). However, the overall prediction performance of this algorithm was lower than that for females, with a prediction 24% better than chance (AUC, 0.62; 95% CI, 0.60-0.62) (Figure 1). Sensitivity was also lower in the model predicting suicide attempt among males (0.32). However, the specificity (0.82), PPV (0.62), and NPV (0.71) for males were similar to the results in the model for females. The 10 top factors were adulthood mother antisocial score, adolescent father antisocial score, mother perceived warmth and affection, adulthood father antisocial score, family size, adolescent mother antisocial score, mother highest level of education, mother depression, mother perceived marital support, and gestational age (Figure 2).

## Discussion

To our knowledge, this population-based prognostic study is the first to examine the value of early life factors for the prediction of suicide attempt in adolescents and young adults in a representative birth cohort. Using a random forest algorithm, a performant machine learning technique, we created classification algorithms predicting suicide attempt from information (ie, 150 potential factors) assessed within the first 5 months of life by both parents, as well as from medical information extracted from hospital birth records. Although the specificity and NPV were acceptable, the AUC, sensitivity, and PPV of the final models suggested a moderate prediction accuracy. More explicitly, this indicates that child, parent, family, and neighborhood characteristics assessed within the first 5 months of life were able to correctly identify 76% to 82% of youths as individuals who would not attempt suicide; that among the overall youth predicted as individuals who would not attempt suicide, 71% to 75% actually would not attempt suicide; that the global prediction of youth suicide attempt was 24% to 44% better than chance; that 32% to 50% of the youths who attempt suicide were correctly identified by the algorithms using information available at age 5 months; and that 60% to 62% of youths identified as individuals who would attempt suicide would actually attempt suicide in adolescence or young adulthood.

### Performance of the Models

The comparison of the performances of our models with those in previous studies is limited by the lack of machine learning studies investigating the ability of early life factors to predict suicide attempt using population samples. However, and not surprisingly, the performance of our models using distal factors was lower compared with studies considering proximal factors.^50,51^ Indeed, the algorithms we developed showed moderate prediction performances, as indicated by the area under the curve and the sensitivity. However, the specificity, NPV, and PPV of the model were acceptable, indicating that among 10 individuals that the algorithm predicted would attempt suicide, at least 6 did actually attempt suicide 2 decades later. In comparison, using proximal factors, clinical samples, and administrative data, previous studies showed AUC values higher than 0.9 for the prediction of suicide attempt in adolescents (vs general hospital controls).^50^ Different factors may explain these relatively modest performances. First, as in most population-based samples, the number of individuals not reporting a suicide attempt outnumbered those reporting a suicide attempt, which is a challenge for prediction, as the algorithm focuses on the larger group and tends to predicts everyone as nonsuicidal.^48^ Although we accounted for this imbalance by applying oversampling techniques, prediction of complex behaviors in the general population remains more difficult than in case-control studies.^52^ Second, our analyses considered factors measured during a very specific time window (ie, perinatal and first months of life), and despite the recognized importance of distal factors associated with suicide attempt, events experienced by age 5 months are insufficient on their own to fully predict outcomes 20 years later. This is illustrated by the sensitivity values: they suggest that, while some individuals are identifiable as at high risk for suicide attempt since early in life, for most individuals, experiences in later stages of life may have a larger influence on suicide risk. This is in line with studies suggesting that factors occurring in middle childhood, such as maltreatment, are important elements in the pathway to suicide,^53^ and studies that highlighted the important role of proximal adolescent factors, such as exposure to bullying victimization, early puberty, and substance use.^5,54^ It is also important to note that predictions were globally better for females than for males. This may be owing to the higher proportion of females in our sample, which provided the algorithm with more individuals to learn from.

### Identified Factors

Despite the low performances of the prediction models, the main identified factors corroborate findings from previous association studies. The main categories of factors identified include socioeconomic and demographic characteristics of the family (eg, mother and father education and age, socioeconomic status, neighborhood characteristics), parents’ psychological state (specifically parents’ antisocial behaviors), and parenting practices. However, some birth-related variables also contributed to the prediction of suicidal behavior (eg, prematurity). These findings add to the existing body of knowledge by showing that early life socioeconomic characteristics and exposure to parental mental and behavioral problems, which have been identified in previous correlational studies as main factors of suicide-related outcomes, are emphasized in predictive models even if their ability to identify youth at risk for suicide attempt is limited.^55,56,57,58,59^

**Figure 3.**
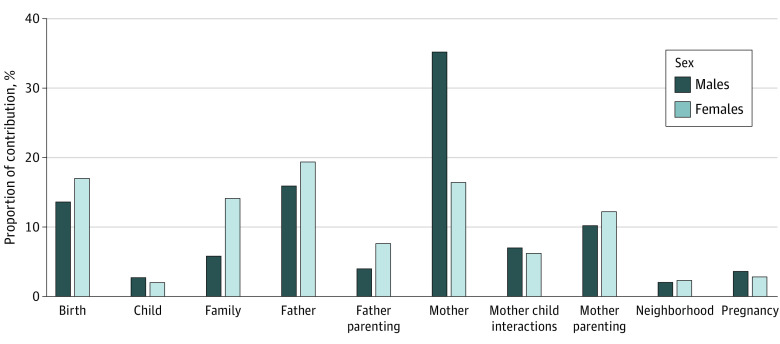
Relative Importance of the 30 Top Factors Identified by the Algorithm Predicting Suicide Attempt The relative contribution of the categories of early life factors to which the 30 top factors belong are depicted. Each factor is weighed according to its contribution to the mean decrease in prediction accuracy.

We identified some common factors for males and females, including parents’ demographic and psychological characteristics (eg, level of education, age at birth, antisocial behavior scores and depression), parenting practices, and perceived neighborhood safety (Figure 3). However, we also found substantial sex differences. Overall, for females, family-related socioeconomic and demographic characteristics (eg, socioeconomic status or family size, maternal and paternal level of education, and age at childbirth) were identified as top factors, while for males, parents’ antisocial behavior and parenting characteristics were identified as top factors.

### Limitations

This study has some limitations. First, owing to attrition, analyses were performed on only 77.6% of participants from the initial representative sample, which calls for caution in the generalizability of the findings to the original population. Second, although we considered a wide range of early life factors, other potentially important factors may have been unmeasured in our cohort, such as parents’ lifetime diagnoses of mental illness and history of suicide attempt. Third, some variables may be affected by measurement bias. For example, self-reported smoking or alcohol use during pregnancy may be influenced by desirability bias yielding conservative estimates; however, our objective was to specifically rely on information obtainable from self-reports by questionnaires or by interview of a perinatal practitioner more than on objective measures. Fourth, we measured recall of the past 12 months for suicidal attempt, thus we potentially missed attempts that occurred at ages 14, 16, 18, and 19 years, thus underestimating our predictions. This bias might have been partially addressed by the lifetime questions at age 20 years.

## Conclusions

The findings of this prognostic study based on innovative machine learning techniques suggest that early life factors previously associated with suicide attempt only modestly contributed to its prediction. Therefore, although those factors may be important in helping us to understand the developmental origins of suicide, their role in the long-term prediction of suicidal behavior is limited. Our findings also stress the importance of later phases of development in the pathway to suicide attempt. Indeed, while youths identified by our algorithm as at risk for suicide attempt in adolescence or young adulthood from early life factors had indeed attempted suicide in adolescence or young adulthood, most youths who attempt suicide are not predicted solely based on early life factors. Although this observation may seem obvious, it stresses the importance of considering observational findings from association studies, often based on very large samples,^48,60^ in a nondeterministic way. Future research should additionally consider the predictive values of early life factors in the contexts of other more proximal risk factors and replicate our findings in other population-based and clinical samples to improve our understanding about the prediction of suicidal behavior.

## Notes

Supplement. **eAppendix.** Details of Statistical Analyses **eTable.** Assessment of Early Life Factors **eReferences**
