# Supplementary material for: Machine Learning Assessment of Early Life Factors Predicting Suicide Attempt in Adolescence or Young Adulthood
Source: JAMA Netw Open. 2021 Mar 12;4(3):e211450. doi: 10.1001/jamanetworkopen.2021.1450 (PMC7955274; doi:10.1001/jamanetworkopen.2021.1450)
Supplement: Supplement. — eAppendix. Details of Statistical Analyses eTable. Assessment of Early Life Factors eReferences [file jamanetwopen-e211450-s001.pdf]

Supplemental Online Content

Navarro MC, Ouellet-Morin I, Geoffroy MC, et al. Machine learning assessment of early life factors predicting suicide attempt in adolescence or young adulthood. *JAMA Netw Open*. 2021;4(3):e211450. doi:10.1001/jamanetworkopen.2021.1450

eAppendix. Details of Statistical Analyses

eTable. Assessment of Early Life Factors

eReferences.

This supplemental material has been provided by the authors to give readers additional information about their work.

© 2021 Navarro MC et al. *JAMA Network Open.*

eAppendix. Details of Statistical Analyses

Random forest approach: We conducted our analyses using a random forest algorithm, a validated machine learning method with good performance and robustness.[1] This technique can be applied to both classification and regression problems and can handle both categorical and continuous predictors. The overall goal of a random forest algorithm is to find the most accurate combination of variables to predict a new observation.

Random forest is a non-parametric ensemble learning method that results from the aggregation of a set of decision trees, created with recursive bootstraps of the initial sample.[2] For each decision tree, a prediction algorithm is created with 2/3 of the subsample, the remaining observations (called out-of-bag sample, OOB) is used to test the performance of the prediction algorithm, measured by the prediction error (called out-of-bag error). Decision trees are created by performing recursive binary splits of the predictor space, containing all the predictor variables, to create sub-spaces called nodes. Each observation goes from the "parent" to the "child" node according to the optimal split value of the predictor variable obtained according to the principle of maximum homogeneity for the outcome in each node. The number of predictor variables used at each node to create the prediction was set at the square root of the total number of predictor variables ($\sqrt{150} \approx 12$), while the number of trees generated by the algorithm was fixed at 1000. All the derived trees are then aggregated to obtain the final prediction model.

To perform the analysis, we randomly split our original dataset into a training and a testing sample. Each predictor was therefore obtained using the training sample (80% of observations), and subsequently validated in the testing sample (20% of observations). To obtain an unbiased estimation of the prediction error, the analyses were repeated several times with different training and testing samples resulting from different random split of the original sample.[3] Considering the important sex differences in mental health symptoms, we conducted separate analyses for boys and girls. Analyses were performed in R with the *randomForest* and *caret* packages.

Variables importance in prediction: Random forests allow one to visualize and quantify the contribution of each variable in the outcome prediction. The OOB samples, which also give the OOB prediction error, are used to calculate these measures. In each OOB sample, the values of a given variable are randomly shifted before applying the initially created prediction algorithm and computing the new prediction error. Then, the difference between the prediction error in the shifted OOB sample and the prediction error in the initial OOB sample is calculated. The magnitude of the increase of the prediction error after shifting the values of the variable is an indication of the importance of the variable in the prediction: a high increase of the prediction errors indicates that the variable is very important for the prediction mode, while a small (or undetectable) change indicates that the variable had a small contribution to the prediction.[4] This process is repeated for each variable in each OOB sample of the forest, so the final variable importance is obtained by averaging the differences between the prediction error in the all shifted OOB samples and the prediction error in the initial OOB samples. The ratio of error is called mean decrease in accuracy (**Figure 2** in the main text) and is unitless. The more a variable is important for the prediction, the higher the mean decrease in accuracy is.

Dealing with unbalanced dataset: As in most population samples, controls outnumbered cases. This unbalanced dataset may bias the prediction algorithm because it will focus only on predicting the majority class, and individuals in the minority class will be incorrectly classified. To deal with this problem, we used the Synthetic Minority Over-sampling Technique (SMOTE) algorithm[5] which creates synthetic data of the smallest class (i.e., symptomatic youth in our sample) based on the n-nearest neighbors method. This technique allows the random forest algorithm to have more symptomatic behavior examples to learn from. Dealing with unbalanced data in challenging for machine learning models, even if generating synthetic data is not as ideal as using a balanced sample, the SMOTE algorithm allows good predictive performance results.[6,7] This was performed using the R package *DMwR*.

Dealing with Missing values: In the original dataset, there was 5% of missing data among the predictor variables. To handle these missing data, we used the nonparametric R *missForest* algorithm to impute missing data.[8]

© 2021 Navarro MC et al. *JAMA Network Open.*

eTable. Assessment of Early Life Factors

	Description
**Birth-related characteristics**	
Birthweight (gr.)	Continuous variable, measured in grams
Duration of pregnancy (weeks)	Continuous variable, measured in weeks
Mother hospital transfer	Mother transferred in specialized hospital (yes/no)
Score for Neonatal Risk	Continuous variable, aggregated index of characteristics indicative of the health conditions of the newborn, range 0-8
APGAR Score 1 minute	Score indicating the global newborn health and adaptation 1 minute after birth. Continuous variable, range 1-10
APGAR Score 5 minutes	Score indicating the global newborn health and adaptation 5 minutes after birth. Continuous variable, range 1-10
Head circumference	Baby head circumference after birth. Continuous variable, measured in centimeters, range 26.5-39 cm
Baby length	Baby size after birth. Continuous variable, measured in centimeters, range 35.5-59 cm
Baby time in hospital	Length of stay of the baby in the hospital after birth. Continuous variable
Birth stimulation	Having received stimulation to go into labor (yes/no)
Duration of labor	Time of delivery. Continuous variable, measured in hours-minutes
Episiotomy	Episiotomy for birth (yes/no)
Induction	Having received induction of labor (yes/no)
Tools during labor	Tools for help to give birth (yes/no)
Fetal presentation before birth	Face presentation of the baby for birth (yes/no)
**Child characteristics**	
Birth order	Continuous variable indicating the rank among the sibling
Number of siblings	Continuous variable
Ethnicity	7 variables (yes/no): Canadian, French, British, European, Amerindian, African, Other
Positives interactions	Score indicating positive parenting practices, rated by external evaluators during home visits with the Home Observation for Measurement of the Environment (HOME). Continuous variable, range 0-10
Attending daycare	Child attended any form of daycare (yes/no)
Daycare type	7 variables (yes/no) indicating the type of daycare: Nursery school, Play group, Day nursery, library, child stimulation program, mother-child program, other
Daycare hours/week	Time per week where the child attends daycare. Continuous variable
Difficult temperament (2 items one for mother, one for father)	Assessment of the child temperament using the Infant Characteristics Questionnaire (7 items).[9] Continuous variables, range 0-10
**Mother-child interactions**	
IMF Simulation	Maternal stimulation of the child, rated by external evaluators during home visits with the Home Observation for Measurement of the Environment (HOME). Continuous variable, range 0-10
IMF Verbalization	Maternal vernal responsiveness to the child, rated by external evaluators during home visits with the HOME. Continuous variable, range 0-10
Positive interactions	Maternal positive interaction with the child, rated by external evaluators during home visits with the HOME. Continuous variable, range 0-10
**Mother and father characteristics**	
Ethnicity	7 variables (yes/no): Canadian, French, British, Amerindian, African, Other
Age	Mother and father age at the survey. Continuous variable (years)
Language spoken at home	Language spoken at home for mother/father: French only, English only, Neither English nor French, English and French, Other
Mother tongue	Parents first language: French, English (not French), Neither English or French
Antisocial behavior in adolescence	Assessed for mother and father with binary questions on 5 different conduct problems in adolescence based on the DSM-IV criteria for conduct disorder and antisocial personality disorder. Continuous variable, range 0-10
Antisocial behavior in adulthood	Assessed for mother and father with binary questions on 5 different conduct problems in adulthood based on the DSM-IV criteria for conduct disorder and antisocial personality disorder. Continuous variable, range 0-10
Highest level of education	Highest level of education achieved by the mother and father (7 response options): Before high school, High school, College, Post high school, Teaching or Communication school, Incomplete university, University
Highest diploma	Highest mother and father diploma: No high school diploma, High school diploma, Post high school diploma, University diploma
Working status at the survey	Mother and father working at the moment of the survey (yes/no)
Working status, past 12 months	Mother and father working in the past 12 months (yes/no)
Type of employment	Type of work status (3 response options): Unemployed, Part-time, Full-time

© 2021 Navarro MC et al. *JAMA Network Open.*

Previous wedding	Mother and father previous wedding (yes/no)
Immigration status	Mother and father immigration status (3 response options): Not immigrant, European immigrant, Non-European immigrant
Years since immigration	Mother and father years since immigration (4 response options): Not immigrant, Less than 5 years, 5 to 9 years, More than 10 years
Depression[10]	Mother and father depression score, assessed using a short version of the Centre for Epidemiological Study Depression Scale. Continuous variable, scale 0-10
Parental parenting: Self-efficacy, impact, hostility–reactivity, warmth, and overprotection[11]	5 variables assessing the following parenting dimensions for mother and father: perceived self-efficacy (6 items), impact (6 items), hostility-reactivity (7 items), warmth/affection (5 items) and overprotection (5 items) to the child. Assessed with the Parental Cognitions and Conduct Toward the Infant Scale. Continuous, range 0-10
Feeling about own health*	General feeling about her own health: Poor, Fair, Good, Very Good, Excellent
Number of abortions*	How often the mother had an abortion. Continuous variable
Smoke during pregnancy*	4 variables related to smoke during pregnancy (yes/no): First trimester, Second trimester, Third trimester, All pregnancy
Number of cigarettes*	Number of cigarettes smoked per day during pregnancy. Continuous variable
Alcohol during pregnancy*	Mother consumed alcohol during pregnancy (7 response options): Never, Less than once per month, 1 to 3 times/months, Once per week, 2 to 3 times/week, 4 to 6 times/week, Every day
Number of drinks*	Usual quantity of alcohol during the pregnancy (4 response options): Zero, 1-2 glasses, 3-4 glasses, More than 5 glasses
Timing of alcohol consumption*	4 variables indicating alcohol consumption (yes/no): First trimester, Second trimester, Third trimester, All pregnancy
Prescribed medications*	4 variables indicating use of prescribed medications (yes/no): First trimester, Second trimester, Third trimester, All pregnancy
Over-the-counter medications*	4 variables indicating use of over-the-counter medications (yes/no): First trimester, Second trimester, Third trimester, All pregnancy
Illegal drugs*	4 variables indicating use of illegal drugs (yes/no): First trimester, Second trimester, Third trimester, All pregnancy
**Family characteristics**	
Family size	Number of persons at home. Continuous variable.
Primary source of income	Main source of income of the household (4 response options): Salary, Self-employment, Welfare, Unemployment insurance, Other
Insufficient household income	Calculated according to Statistics Canada's guidelines and categorized into: Sufficient, Insufficient, Very insufficient
Socioeconomic status	Continuous variable, aggregation of 5 items (e.g. parental education, occupation and annual gross income), range -3;3 and 0 centered.
Family type	2 items family structure at the survey and at birth (3 response options): Intact, Always single parent, Widowed
Single-parent family	Baby birth in a single-parent family (yes/no)
Biological parents at home	2 variables (yes/no): both biological parents; biological father living at home
Marital status at childbirth	Parents marital status at birth (5 response options): Married; Common Law, Common law but married later, Separated, Never lived together
Period of relationship before birth	Time between relationship starts and birth in months. Continuous variable
Family functioning[12]	Assessed with 7 items (eg, do not get along well together) from McMaster Family assessment administered to the mother. Continuous variable, range 0-10 (high scores reflect high dysfunction)
Language spoken at home	Language spoken at home by parents (5 response options): Only French, Only English, Neither French nor English, French and English, French or English +another language
**Neighborhood characteristics**	
Dangerous neighborhood	Measured using 7 items from the Simcha-Fagan Neighbourhood Questionnaire.[13] Continuous variable, range 0-10
Social problems in the neighborhood	Measured using 6 items from the Simcha-Fagan Neighbourhood Questionnaire.[13] Continuous variable, range 0-10

All variables were reported by the person most knowledgeable about the child (mother in 98% of the cases), except when otherwise specified; [a] Extracted from the birth registry; *Answered only by the mothers

© 2021 Navarro MC et al. *JAMA Network Open.*

eReferences

1. Kaur A, Kaur K. An Empirical Study of Robustness and Stability of Machine Learning Classifiers in Software Defect Prediction. In: El-Alfy E-SM, Thampi SM, Takagi H, Piramuthu S, Hanne T, eds. *Advances in Intelligent Informatics*. Advances in Intelligent Systems and Computing. Springer International Publishing; 2015:383-397. doi:10.1007/978-3-319-11218-3_35

2. Breiman L. Random Forests. *Machine Learning*. 2001;45(1):5-32. doi:10.1023/A:1010933404324

3. Poldrack RA, Huckins G, Varoquaux G. Establishment of Best Practices for Evidence for Prediction: A Review. *JAMA Psychiatry*. Published online November 27, 2019. doi:10.1001/jamapsychiatry.2019.3671

4. Genuer R, Poggi J-M, Tuleau-Malot C. Variable selection using random forests. *Pattern Recognition Letters*. 2010;31(14):2225-2236. doi:10.1016/j.patrec.2010.03.014

5. Chawla NV, Bowyer KW, Hall LO, Kegelmeyer WP. SMOTE: Synthetic Minority Over-sampling Technique. *Journal of Artificial Intelligence Research*. 2002;16:321-357. doi:10.1613/jair.953

6. Amin A, Anwar S, Adnan A, et al. Comparing Oversampling Techniques to Handle the Class Imbalance Problem: A Customer Churn Prediction Case Study. *IEEE Access*. 2016;4:7940-7957. doi:10.1109/ACCESS.2016.2619719

7. Teh K, Armitage P, Tesfaye S, Selvarajah D, Wilkinson ID. Imbalanced learning: Improving classification of diabetic neuropathy from magnetic resonance imaging. *PLOS ONE*. 2020;15(12):e0243907. doi:10.1371/journal.pone.0243907

8. Stekhoven DJ, Bühlmann P. MissForest—non-parametric missing value imputation for mixed-type data. *Bioinformatics*. 2012;28(1):112-118. doi:10.1093/bioinformatics/btr597

9. Bates JE, Freeland CAB, Lounsbury ML. Measurement of Infant Difficultness. *Child Development*. 1979;50(3):794-803. doi:10.2307/1128946

10. Lewinsohn PM, Seeley JR, Roberts RE, Allen NB. Center for Epidemiologic Studies Depression Scale (CES-D) as a screening instrument for depression among community-residing older adults. *Psychol Aging*. 1997;12(2):277-287.

11. Boivin M, Pérusse D, Dionne G, et al. The genetic-environmental etiology of parents' perceptions and self-assessed behaviours toward their 5-month-old infants in a large twin and singleton sample. *Journal of Child Psychology and Psychiatry*. 2005;46(6):612-630. doi:10.1111/j.1469-7610.2004.00375.x

12. Ontario Child Health Study: Reliability and Validity of the General Functioning Subscale of the McMaster Family Assessment Device - BYLES - 1988 - Family Process - Wiley Online Library. Accessed October 8, 2019. https://onlinelibrary.wiley.com/doi/full/10.1111/j.1545-5300.1988.00097.x

13. McGuire JB. The reliability and validity of a questionnaire describing neighborhood characteristics relevant to families and young children living in urban areas. *Journal of Community Psychology*. 1997;25(6):551-566. doi:10.1002/(SICI)1520-6629(199711)25:6<551::AID-JCOP5>3.0.CO;2-S

© 2021 Navarro MC et al. *JAMA Network Open.*
